# Quantitative of progesterone using isotope dilution-matrix-assisted laser desorption ionization-time of flight mass spectrometry

**DOI:** 10.1016/j.mex.2020.100812

**Published:** 2020-02-20

**Authors:** Ming-Hui Yang, Han-Ping You, Hsin-Yi Wu, Yi-Ming Arthur Chen, Ying-Fong Huang, Yu-Chang Tyan

**Affiliations:** a National Mosquito-Borne Diseases Control Research Center, National Health Research Institutes, Miaoli, Taiwan; b Clinical Pharmacogenomics and Pharmacoproteomics, College of Pharmacy, Taipei Medical University, Taipei, Taiwan; c Department of Medical Imaging and Radiological Sciences, Kaohsiung Medical University, Kaohsiung, Taiwan; d Instrumentation Center, National Taiwan University, Taipei, Taiwan; e Department of Nuclear Medicine, Kaohsiung Medical University Hospital, Kaohsiung, Taiwan; f Graduate Institute of Medicine, College of Medicine, Kaohsiung Medical University, Kaohsiung 807, Taiwan; g Institute of Medical Science and Technology, National Sun Yat-sen University, Kaohsiung 804, Taiwan; h Department of Medical Research, Kaohsiung Medical University Hospital, Kaohsiung 807, Taiwan; i Research Center for Environmental Medicine, Kaohsiung Medical University, Kaohsiung 807, Taiwan; j Center for Cancer Research, Kaohsiung Medical University, Kaohsiung, Taiwan

**Keywords:** MALDI-TOF/MS, Progesterone, Isotope dilution

## Abstract

A quantification assay based on isotope dilution mass spectrometry to determine the concentration of progesterone in human serum was reported. Incorporated with ^13^C_3_-progesterone, serum samples were subjected to progesterone extraction and clean-up by C4 solid-phase-extraction columns and hexane-based liquid/liquid extraction, respectively. The cleaned-up serum samples were then subjected to MALDI-TOF mass spectrometry for the quantification of progesterone. In the study, the recovered progesterone concentration determined by the assay showed good robustness and constancy in comparison to conventional radioimmunologic assay. We concluded that the ^13^C_3_-progesterone-based quantification assay is a robust method for the measurement of serum progesterone.

Advantages of this technique includes:

• This study describes a MALDI-TOF/MS method for the determination of serum progesterone.

• The technique is simple and easy to apply on MALDI-TOF/MS for serum progesterone analysis.

• The correlation coefficient between MALDI-TOF MS and RIA was 0.981 for serum progesterone.

## Graphical abstract

**Figure:**
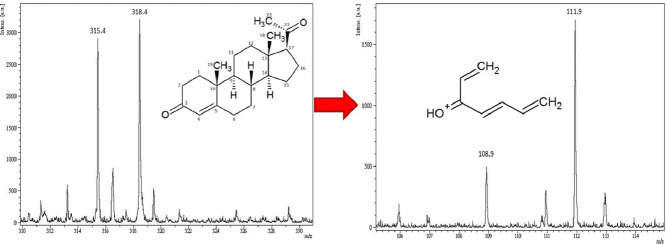

**Table:** Specifications table

Subject Area:	*Biochemistry, Genetics and Molecular Biology*
More specific subject area:	Quantification assay of serum progesterone
Method name:	Quantitative of progesterone
Name and reference of original method:	Quantitative analysis of progesterone using isotope dilution-matrix-assisted laser desorption ionization-time of flight mass spectrometry as a reference procedure for radioimmunoassay https://doi.org/10.1016/j.cca.2019.11.020
Resource availability:	

## Method details

### Required equipment

The MOLDI-TOF/MS used in the study was the model Autoflex III Smartbeam with nitrogen laser (VSL-337, 337 nm), which is manufactured by Bruker Inc., USA. Data of the mass spectra was collected in the reflector positive-ion mode with 25-kV acceleration voltage and 300-ns delay. The grid and guide wire voltages were set to be 90.0 and 0.15%, respectively.

### Serum sample preparation

(1) Human serum was collected in sterile tubes and centrifuged at 1000 *g* for 10 min at 4 °C.
(2) 1 mL of the supernatant was adjusted to pH 9.8 ± 0.2 with 0.1 g/mL carbonate/bicarbonate buffer [1].
(3) An incorporation of ^13^C_3_-Progesterone (15 ng/mL, Sigma-Aldrich, USA) was added as the internal standard isotope.

### Progesterone extraction

(1) The serum sample was subjected to a methanol and double-distilled water pre-equilibrated solid phase extraction column (SPE Supra-Clean 300 Å C4, PerkinElmer, USA) for the extraction of relatively hydrophobic progesterone.
(2) Passing the sample through the column and discarding the flow-through, the progesterone capturing column was washed with 2 mL double-distilled water.
(3) The captured progesterone was then eluted with 4 mL of methanol.
(4) The eluates were subjected to centrifugal evaporation to remove the solvent.

### Serum progesterone clean-up

(1) The progesterone extracted from the serum was dissolved in 1 mL of 0.2 M pH 9.8 ± 0.2 carbonate buffer.
(2) 2.5 mL of hexane was added to the test tube, and the tube was subjected to vigorous shaking for 20 min [2,3].
(3) After centrifugation at 2000 rpm for 5 min, the tube was incubated at −20 °C for phase separation.
(4) Discarding the frizzed lower-phase, the supernatant was transferred to a new tube for solvent evaporation by nitrogen gas.
(5) The cleaned-up serum progesterone sample was finally dissolved in 5 µL of 50% ethanol prior to MALDI-TOF/MS analysis.

### Sample preparation for mass spectrum

(1) 5 µL of each serum progesterone sample described above was mixed with 1 µL of the matrix, 2,5-Dihydroxybenzoic acid (DHB, Sigma-Aldrich, USA) [4].
(2) Deposited on the sample tray at room temperature. After the sample-matrix mix was dried, the tray was subjected to measurement and generally 100 laser shots were used in the analytical process. All the samples were measured in triplicate.

**Fig. 1:**
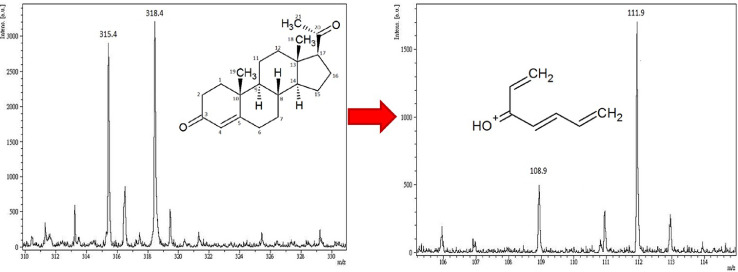
MALDI-TOF MS spectrum of progesterone and ^13^C_3_-progesterone. Representative peaks at m/z 108.9 and 111.9 were obtained for progesterone and ^13^C_3_-progesterone (asterisk denotes ^13^C).

In the detection of progesterone in human serum samples, the signal (*m/z* 108.9) of a particular progesterone fragment was specifically used to resemble the amount of progesterone and to minimize the interference resulting from other compounds present in serum, which were co-extracted by the C4 SPE and hexane extraction [5]. A representative MALDI-TOF/MS spectrum showing the peak of fragmented serum progesterone (*m/z* = 108.9) and the isotopic standard (*m/z* = 111.9) is shown in Fig. 1.
